# Supplementary material for: Polyketide synthase gene clusters in the ericoid mycorrhizal fungus Oidiodendron maius: genome-wide prediction, expression profiling, and functional characterization of knockout mutants
Source: Front Plant Sci. 2026 Sep 16;17:1924430. doi: 10.3389/fpls.2026.1924430 (PMC13623903; doi:10.3389/fpls.2026.1924430)
Supplement: Supplementary file 1 [file SupplementaryFile1.zip › Supplementary Materials/Supplementary Folder 1.zip!/Oidma1_AssemblyScaffolds_Repeatmasked/knownclusterblast/cluster37/jgi.p_Oidma1_132500_mibig_hits.html]

| MIBiG Protein | Description | MIBiG Cluster | MiBiG Product | % ID | % Coverage | BLAST Score | E-value |
| --- | --- | --- | --- | --- | --- | --- | --- |
| ACU71635.1 | short-chain\_dehydrogenase/reductase\_SDR | BGC0001154 | Cacibiocin\_biosynthetic\_gene\_cluster | 32 | 97.628458498 | 113 | 3e-25 |
| CAM59605.1 | putative\_reductase | BGC0000297 | Aeruginoside\_biosynthetic\_gene\_cluster | 30 | 100.395256917 | 112 | 8.7e-25 |
| AFO85456.1 | reductase/oxidase | BGC0000391 | Methylpendolmycin/pendolmycin\_biosynthetic\_gene\_cluster | 34 | 100.0 | 111 | 1.1e-24 |
| ACM68688.1 | AerF | BGC0000298 | Aeruginosin\_biosynthetic\_gene\_cluster | 30 | 99.604743083 | 108 | 1.6e-23 |
| EDY42534.1 | monensin\_polyketide\_synthase\_ketoacyl\_reductase | BGC0000212 | Cinerubin\_B\_biosynthetic\_gene\_cluster | 30 | 100.0 | 107 | 2.1e-23 |
| ABC42552.1 | putative\_3-ketoacyl\_ACP\_reductase | BGC0000698 | Hygromycin\_A\_biosynthetic\_gene\_cluster | 31 | 98.814229249 | 106 | 6.3e-23 |
| AFJ52673.1 | C-9\_ketoreductase | BGC0001073 | Kosinostatin\_biosynthetic\_gene\_cluster | 31 | 100.0 | 106 | 6.3e-23 |
| ADD83006.1 | PtnO8 | BGC0001156 | Platencin\_biosynthetic\_gene\_cluster | 30 | 101.581027668 | 105 | 1.1e-22 |
| AEI98650.1 | CtcG | BGC0000209 | Chlortetracycline\_biosynthetic\_gene\_cluster | 30 | 100.0 | 104 | 2.4e-22 |
| CAG14968.1 | ketoreductase | BGC0000253 | Oviedomycin\_biosynthetic\_gene\_cluster | 30 | 100.0 | 104 | 2.4e-22 |
| AAT12286.1 | LtxD | BGC0000384 | Lyngbyatoxin\_biosynthetic\_gene\_cluster | 34 | 99.209486166 | 103 | 3.1e-22 |
| ACO31295.1 | PtmO8 | BGC0001140 | Platensimycin\_/\_platencin\_biosynthetic\_gene\_cluster | 30 | 101.581027668 | 102 | 6.9e-22 |
| ABS75236.1 | DhbA | BGC0001185 | Bacillibactin\_biosynthetic\_gene\_cluster | 29 | 100.790513834 | 101 | 1.5e-21 |
| AGH68923.1 | oxidoreductase | BGC0001083 | Merochlorin\_biosynthetic\_gene\_cluster | 34 | 101.185770751 | 99 | 4.5e-21 |
| AHL46698.1 | ketoreductase | BGC0001177 | Mensacaricin\_biosynthetic\_gene\_cluster | 29 | 100.0 | 98 | 1e-20 |
| ABP73642.1 | SalM | BGC0000145 | Salinosporamide\_A\_biosynthetic\_gene\_cluster | 28 | 97.628458498 | 97 | 1.7e-20 |
| ABP53501.1 | short-chain\_dehydrogenase/reductase\_SDR | BGC0001041 | Salinosporamide\_biosynthetic\_gene\_cluster | 28 | 97.628458498 | 97 | 1.7e-20 |
| CAJ42322.1 | ketoreductase | BGC0000273 | Steffimycin\_biosynthetic\_gene\_cluster | 34 | 98.814229249 | 97 | 1.7e-20 |
| AAQ08912.1 | putative\_3-ketoacyl-ACP\_reductase | BGC0000224 | Fredericamycin\_biosynthetic\_gene\_cluster | 34 | 101.185770751 | 97 | 2.2e-20 |
| AGO50613.1 | ketoreductase | BGC0000229 | Grincamycin\_biosynthetic\_gene\_cluster | 29 | 101.185770751 | 97 | 2.2e-20 |
| BAB72043.1 | AknA | BGC0000191 | Aclacinomycin\_biosynthetic\_gene\_cluster | 29 | 100.0 | 96 | 6.5e-20 |
| BAV17004.1 | putative\_oxygenase-reductase | BGC0001384 | Saprolmycin\_biosynthetic\_gene\_cluster | 33 | 102.766798419 | 95 | 1.1e-19 |
| CAQ64702.1 | probable\_glucose\_dehydrogenase | BGC0000087 | Lasalocid\_biosynthetic\_gene\_cluster | 30 | 99.604743083 | 94 | 1.4e-19 |
| WP\_030269276.1 | short-chain\_dehydrogenase | BGC0001308 | Simocyclinone\_D9\_/\_simocyclinone\_D10\_/\_simocyclinone\_D11 | 31 | 102.766798419 | 94 | 1.9e-19 |
| AHL46733.1 | ketoreductase | BGC0001179 | Rishirilide\_B\_biosynthetic\_gene\_cluster | 28 | 100.0 | 93 | 4.2e-19 |
| KDN80052.1 | ketoreductase | BGC0001074 | Cosmomycin\_D\_biosynthetic\_gene\_cluster | 31 | 97.628458498 | 92 | 7.2e-19 |
| ADG86311.1 | 3-ketoacyl\_ACP-reductase | BGC0000190 | A-74528\_biosynthetic\_gene\_cluster | 31 | 99.209486166 | 92 | 7.2e-19 |
| AFV52172.1 | ketoreductase | BGC0000081 | Kedarcidin\_biosynthetic\_gene\_cluster | 30 | 97.628458498 | 92 | 7.2e-19 |
| BAH33405.1 | 2,3-dihydro-2,3-dihydroxybenzoate\_dehydrogenase | BGC0000371 | Heterobactin\_biosynthetic\_gene\_cluster | 28 | 102.766798419 | 92 | 9.3e-19 |
| AAK57530.1 | PgaM | BGC0000262 | Rabelomycin\_biosynthetic\_gene\_cluster | 32 | 103.162055336 | 91 | 1.2e-18 |
| AGO50615.1 | oxygenase-reductase | BGC0000229 | Grincamycin\_biosynthetic\_gene\_cluster | 33 | 102.766798419 | 91 | 1.2e-18 |
| CBF90105.1 | oxidoreductase,\_putative\_(Eurofung) | BGC0001271 | Emericellin\_biosynthetic\_gene\_cluster | 27 | 100.395256917 | 91 | 1.6e-18 |
| ABZ01830.1 | ESC\_reductase | BGC0001272 | Elsinochrome\_B\_/\_elsinochrome\_C\_biosynthetic\_gene\_cluster | 27 | 99.604743083 | 91 | 1.6e-18 |
| CAH10114.1 | putative\_ketoreducatse | BGC0000268 | Sch47554\_/\_Sch47555\_biosynthetic\_gene\_cluster | 27 | 100.0 | 91 | 1.6e-18 |
| CAA34368.1 |  | BGC0000228 | Granaticin\_biosynthetic\_gene\_cluster | 29 | 101.185770751 | 91 | 1.6e-18 |
| CAA09652.1 | polyketide\_ketoreductase | BGC0000227 | Granaticin\_biosynthetic\_gene\_cluster | 29 | 101.185770751 | 91 | 1.6e-18 |
| AAX57196.2 | auricin\_polyketide\_oxygenase-reductase | BGC0000201 | Auricin\_biosynthetic\_gene\_cluster | 30 | 102.766798419 | 91 | 2.1e-18 |
| ADI71448.1 | putative\_oxygenase-reductase | BGC0000203 | BE-7585A\_biosynthetic\_gene\_cluster | 32 | 102.766798419 | 90 | 2.7e-18 |
| CAH10112.1 | putative\_oxidase-reducatse | BGC0000268 | Sch47554\_/\_Sch47555\_biosynthetic\_gene\_cluster | 32 | 101.185770751 | 90 | 2.7e-18 |
| CAA60458.1 | ketoreductase/dehydrogenase | BGC0001040 | Rapamycin\_biosynthetic\_gene\_cluster | 30 | 104.743083004 | 89 | 4.6e-18 |
| BAJ52708.1 | putative\_3-ketoacyl-ACP\_reductase | BGC0000222 | FD-594\_biosynthetic\_gene\_cluster | 32 | 98.023715415 | 88 | 1e-17 |
| AFY23045.1 | TamN | BGC0000274 | Tetarimycin\_biosynthetic\_gene\_cluster | 27 | 98.814229249 | 87 | 1.8e-17 |
| AIL50168.1 | putative\_ketoreductase | BGC0000213 | Colabomycin\_biosynthetic\_gene\_cluster | 29 | 99.604743083 | 87 | 3e-17 |
| CAD62198.1 | Ata4\_protein | BGC0000873 | A201A\_biosynthetic\_gene\_cluster | 30 | 99.604743083 | 86 | 3.9e-17 |
| AHA81975.1 | Arm5 | BGC0000199 | Arimetamycin\_biosynthetic\_gene\_cluster | 32 | 98.814229249 | 86 | 3.9e-17 |
| ADB02849.1 | AzicF | BGC0000202 | Azicemicin\_biosynthetic\_gene\_cluster | 29 | 101.185770751 | 86 | 5.1e-17 |
| ANY57964.1 | MtcE\_3-ketoacyl-ACP\_reductase | BGC0001369 | Metatricycloene\_biosynthetic\_gene\_cluster | 30 | 101.976284585 | 86 | 6.7e-17 |
| AAQ84160.1 | ChcA | BGC0000123 | Phoslactomycin\_B\_biosynthetic\_gene\_cluster | 27 | 101.185770751 | 86 | 6.7e-17 |
| AAK06790.1 | putative\_reductase\_SimA9 | BGC0001072 | Simocyclinone\_biosynthetic\_gene\_cluster | 29 | 102.766798419 | 85 | 8.7e-17 |
| AAL15586.1 | Sim8 | BGC0000270 | Simocyclinone\_biosynthetic\_gene\_cluster | 29 | 102.766798419 | 85 | 8.7e-17 |
| EHM27510.1 | monooxygenase\_FAD-binding | BGC0000235 | Kiamycin\_biosynthetic\_gene\_cluster | 32 | 103.95256917 | 84 | 1.5e-16 |
| ACX35428.1 | BacC | BGC0000888 | Bacilysin\_biosynthetic\_gene\_cluster | 26 | 98.418972332 | 84 | 1.5e-16 |
| ABB52548.1 | 3-oxoacyl-(acyl-carrier-protein)-reductase | BGC0000047 | Dihydrochalcomycin\_biosynthetic\_gene\_cluster | 29 | 100.395256917 | 84 | 2.5e-16 |
| ANC94959.1 | AlmK | BGC0001396 | Aldgamycin\_biosynthetic\_gene\_cluster | 29 | 100.395256917 | 83 | 4.3e-16 |
| ABS75820.1 | BacC | BGC0001184 | Bacilysin\_biosynthetic\_gene\_cluster | 26 | 98.418972332 | 81 | 1.3e-15 |
| AAY93361.1 | glucose\_1-dehydrogenase | BGC0000413 | Pyoverdine\_biosynthetic\_gene\_cluster | 26 | 102.371541502 | 81 | 1.3e-15 |
| AAF00206.1 | oxygenase-reductase | BGC0000276\_c2 | Urdamycin\_biosynthetic\_gene\_cluster | 30 | 100.790513834 | 81 | 2.2e-15 |
| ACJ24879.1 | putative\_short-chain\_dehydrogenase/reductase | BGC0000119 | Pactamycin\_biosynthetic\_gene\_cluster | 30 | 101.185770751 | 78 | 1.1e-14 |
| BAF92605.1 | putative\_short-chain\_dehydrogenase/reductase | BGC0000118 | Pactamycin\_biosynthetic\_gene\_cluster | 30 | 101.185770751 | 78 | 1.1e-14 |
| AAM97360.1 | RubJ | BGC0000266 | Rubromycin\_biosynthetic\_gene\_cluster | 30 | 104.743083004 | 74 | 1.5e-13 |
| AAD32718.1 | MitF | BGC0000915 | Mitomycin\_biosynthetic\_gene\_cluster | 26 | 98.814229249 | 72 | 5.9e-13 |
| AAM54105.1 | glucose\_1-dehydrogenase | BGC0000020 | Ansamitocin\_biosynthetic\_gene\_cluster | 31 | 73.5177865613 | 72 | 1e-12 |
| AAW85354.1 | 3-oxoacyl-(acyl-carrier-protein)\_reductase,\_putative | BGC0000837 | APE\_Vf\_biosynthetic\_gene\_cluster | 26 | 99.604743083 | 70 | 2.9e-12 |
| AFJ52671.1 | ketoreductase | BGC0001073 | Kosinostatin\_biosynthetic\_gene\_cluster | 26 | 97.233201581 | 70 | 3.8e-12 |
| AAD37452.1 | putative\_ketoacyl\_reductase | BGC0000252 | Nonactin\_biosynthetic\_gene\_cluster | 25 | 98.023715415 | 69 | 5e-12 |
| AME18007.1 | oxidoreductase | BGC0001378 | Tiancimycin\_biosynthetic\_gene\_cluster | 28 | 74.7035573123 | 66 | 7.2e-11 |
| CDG76955.1 | 2,3-dihydro-2,3-dihydroxybenzoate\_dehydrogenase | BGC0000446 | Tilivalline\_biosynthetic\_gene\_cluster | 27 | 101.185770751 | 63 | 3.6e-10 |
| CAM34373.1 | putative\_reductase | BGC0000242 | Lysolipin\_biosynthetic\_gene\_cluster | 27 | 101.185770751 | 62 | 1e-09 |
| AAM33669.1 | putative\_3-oxoacyl-ACP\_reductase\_GrhO2 | BGC0000230 | Griseorhodin\_biosynthetic\_gene\_cluster | 27 | 101.976284585 | 62 | 1e-09 |
| ACO31293.1 | PtmO7 | BGC0001140 | Platensimycin\_/\_platencin\_biosynthetic\_gene\_cluster | 26 | 105.138339921 | 61 | 1.4e-09 |
| ADD83005.1 | PtnO7 | BGC0001156 | Platencin\_biosynthetic\_gene\_cluster | 28 | 77.4703557312 | 59 | 5.1e-09 |
| BAR73014.1 | putative\_3-ketoacyl-ACP\_reductase | BGC0001194 | Hitachimycin\_biosynthetic\_gene\_cluster | 24 | 107.509881423 | 56 | 5.7e-08 |
| CUW01160.1 | putative\_3-ketoacyl-(acyl-carrier-protein)\_reductase | BGC0001359 | PM100117\_/\_PM100118\_biosynthetic\_gene\_cluster | 26 | 70.7509881423 | 55 | 1.3e-07 |
| CAM34348.1 | putative\_short-chain\_dehydrogenase/reductase\_SDR | BGC0000242 | Lysolipin\_biosynthetic\_gene\_cluster | 31 | 73.9130434783 | 50 | 2.4e-06 |
